# Pharmacokinetics and Pharmacodynamic Effect of a Blood-Brain Barrier-Crossing Fusion Protein Therapeutic for Alzheimer’s Disease in Rat and Dog

**DOI:** 10.1007/s11095-022-03285-z

**Published:** 2022-06-15

**Authors:** Etienne Lessard, Kerry Rennie, Arsalan Haqqani, Binbing Ling, James Whitfield, Andrea Paradis, Joseph Araujo, Nathan Yoganathan, John Gillard, Danica Stanimirovic, Balu Chakravarthy

**Affiliations:** 1 Human Health Therapeutics, National Research Council, Ottawa, Ontario K1A 0R6 Canada; 2 InterVivo Solutions Inc, Fergus, Ontario Canada; 3 KalGene Pharmaceuticals, Montreal, Québec Canada

**Keywords:** Alzheimer’s disease, blood-brain barrier-crossing biologics, CNS exposure, pharmacokinetics, pharmacodynamics

## Abstract

**Purpose:**

We have recently demonstrated the brain-delivery of an Amyloid-ß oligomer (Aßo)-binding peptide-therapeutic fused to the BBB-crossing single domain antibody FC5. The bi-functional fusion protein, FC5-mFc-ABP (KG207-M) lowered both CSF and brain Aß levels after systemic dosing in transgenic mouse and rat models of Alzheimer’s disease (AD). For development as a human therapeutic, we have humanized and further engineered the fusion protein named KG207-H. The purpose of the present study was to carry out comparative PK/PD studies of KG207-H in wild type rat and beagle dogs (middle-aged and older) to determine comparability of systemic PK and CSF exposure between rodent species and larger animals with more complex brain structure such as dogs.

**Method:**

Beagle dogs were used in this study as they accumulate cerebral Aß with age, as seen in human AD patients, and can serve as a model of sporadic AD. KG207-H (5 to 50 mg/kg) was administered intravenously and serum and CSF samples were serially collected for PK studies and to assess target engagement. KG207-H and Aβ levels were quantified using multiplexed selected reaction monitoring mass spectrometry.

**Results:**

After systemic dosing, KG207-H demonstrated similar serum pharmacokinetics in rats and dogs. KG207-H appeared in the CSF in a time- and dose-dependent manner with similar kinetics, indicating CNS exposure. Further analyses revealed a dose-dependent inverse relationship between CSF KG207-H and Aß levels in both species indicating target engagement.

**Conclusion:**

This study demonstrates translational attributes of BBB-crossing Aβ-targeting biotherapeutic KG207-H in eliciting a pharmacodynamic response, from rodents to larger animal species.

**Supplementary Information:**

The online version contains supplementary material available at 10.1007/s11095-022-03285-z.

## INTRODUCTION

The clinical success of biologics for treating many peripheral diseases has not translated to CNS indications owing at least in part to the difficulty in delivering large biotherapeutics across the blood-brain barrier (BBB) (1). The vast majority of biologics with CNS targets that have entered clinical trials have not been engineered to cross the BBB, potentially contributing to lack of efficacy and highlighting the need to incorporate a BBB delivery strategy into drug development for CNS-targeting therapeutics (2).

A promising strategy for delivering therapeutics across the BBB is to exploit the presence of receptors expressed by the brain endothelium, whose physiological role is to enable transport of endogenous protein ligands from the circulation into the brain via a receptor mediated transcytosis (RMT) pathway. Antibodies targeted to receptors involved in RMT can be used as molecular Trojan horses to carry therapeutic payloads across the BBB (3–5).

FC5 is a camelid single-domain antibody fragment (V_H_H) selected by functional screening of a phage-displayed llama single domain antibody library for its ability to bind and internalize into human brain endothelial cells (BEC) (6). FC5 acts as a ligand for TMEM30a (cdc50a), the beta subunit of a phospholipid ATPase flippase (7). Upon entry into BEC, FC5 is sorted into early endosomes and multivesicular bodies thereby escaping late endosomes/lysosomes, and is released at the abluminal side of the BBB (8, 9).

FC5 is capable of crossing BBB models *in vitro* composed of immortalized rat brain endothelial cells (SV-ARBECs), and human primary or iPSC-derived brain endothelial cells (6, 10–12). Transport of biologic cargo to the CNS *in vivo* is facilitated by FC5, yielding CSF/serum ratios for FC5 fusion proteins greater than that observed for typical antibodies (10, 11, 13). Notably, parenchymal delivery of a full IgG by FC5 after IV injection was confirmed by demonstrating that >90% of antibody concentration measured in the total brain homogenate was found in vessel-depleted brain parenchymal fraction (13), indicating that the fusion protein was not simply trapped in the vasculature but had gained access to the brain tissue. Furthermore, delivery of either the neuropeptide dalargin (10) or a full IgG mGluR1 antagonist (13) to the rodent brain by FC5 elicited a pharmacological response (inhibition of thermal hyperalgesia), supporting the utility of FC5 in transporting functional payloads to their target sites in the CNS.

More recently we have generated a bifunctional fusion protein in which FC5 was fused to an amyloid-β binding peptide (ABP) via mouse IgG2a Fc fragment (FC5-mFc2a-ABP; KG207-M). ABP preferentially binds toxic oligomeric Aβ [believed to be a major culprit in AD (14)], prevents Aβ-induced death of SH-SY5Y neuroblastoma cells and labels Aβ aggregates in human AD brain tissue sections and transgenic APPswe/PSEN1dE9 mouse brain (15, 16).

In aged transgenic McGill-R-Thy1-APP rats expressing human APP_751_ with familial AD mutations, 5 week treatment with KG207-M markedly reduced brain Aβ levels measured by positron emission tomography, reversed hippocampal atrophy, and improved resting state functional connectivity (17). CSF concentration of KG207-M over time was negatively correlated with levels of CSF Aβ quantified by Selected Reaction Monitoring (SRM) mass spectrometry.

The ability of FC5 to deliver biologics to the CNS of species with larger and more complex brains has not been assessed previously. Here we address the translatability of CSF/serum pharmacokinetics of a humanized version of KG207 (KG207-H) from rat to a larger species, Beagle dog. Beagle dogs were chosen because they are commonly used for both PK and toxicology studies (18). More relevant to the present study, dogs are known to spontaneously develop cognitive deficit with age, as well as certain neuropathological hallmarks seen in aged human brain, particularly in the brains of AD patients (19, 20). These include Aβ pathology, reduced brain volume, neuronal loss, and impaired neurogenesis (20, 21). In addition, several Aβ species, including Aβ40 and Aβ42, with an identical sequence to humans (22, 23) are also present in dog’s cerebrospinal fluid (CSF) and their profiles are similar between the canine and human CSF (18, 24). In humans, a decreased CSF Aβ42 to Aβ40 ratio is considered a pathophysiological biomarker of AD. A similar age-dependent decrease in the ratio of Aβ42/Aβ40 is also seen in the CSF of aging Beagle dogs which correlates with brain accumulation of amyloid deposits (18, 25, 26). Collectively, these studies strongly support the utility of dog as an appropriate and natural animal model for research in Alzheimer’s disease and for translational studies of therapeutic drugs that may complement work in rodents and non-human primates (18, 24). Therefore, use of dogs in the current study enables both an assessment of translation of serum/CSF PK of KG207-H from rodent to a larger species, and evaluation of target engagement (changes in CSF Aβ) in dog CNS. The study confirms the cross-species translatability of both the pharmacokinetic and pharmacodynamic profile of KG207-H from small rodents with less complex brain to larger animal species such as dogs with more structurally and functionally complex brain.

## MATERIALS AND METHODS

### Protein Production

KG207-H, a fusion protein comprising humanized BBB carrier FC5 V_H_H fused to N-terminus of human IgG1 Fc fragment and fused to Aß-binding peptide (ABP) at its C-terminus [FC5(H3)-hFc-ABP], was designed and expressed in CHO cells. The expressed protein was purified on MabSelect Sure affinity columns and further purified by cation exchange (CEX) chromatography.

### FC5 Binding to Dog Endothelial Cells *In Vitro*

The cross species binding of FC5-hFc in endothelial cells was assessed by mirrorball*®* high sensitivity microplate cytometry (SPT Labtech Ltd., Melbourn, UK) using protocols published previously (27). Immortalized human (HCMEC/D3/HBEC-D3, Dr. Pierre Olivier Couraud, Cochin Institute Université Paris Descartes INSERM, Ref 28), rat microvascular brain endothelial cells (sv-ARBEC) and Canine Aortic Endothelial Cells (CnAOEC, Cell Applications Inc., San Diego, CA, USA) were cultured until confluent at 37°C in a humidified 5% CO2 atmosphere in T-75 flasks coated with 100 μg/ml rat tail collagen type 1 (BD Canada, Mississauga, ON, Canada).

SV-ARBEC cells were grown in M199 medium supplemented with peptone, D-glucose, BME Amino Acids, BME vitamins, antibiotic/antimycotic and fetal bovine serum (ThermoFisher, Mississauga, ON, Canada). HBEC-D3 cells were cultured in EBM2 basal media (Lonza, Walkersville, MD, USA CC3156) supplemented with one quarter of a SingleQuot kit (Lonza CC 4176) and 2% fetal bovine serum. CnAOEC were cultured in Canine EC Growth Media All in One (Cell Applications, Inc). The cells were dissociated with Trypsin/EDTA (ThermoFisher) and washed in complete M199 serum media. Cell number was calculated using a Bio-Rad TC20 automated cell counter with Trypan Blue dye to assess viability, diluted to 8.0 × 10^4^ cells/ml in appropriate growing media. 50 μl of each cell suspension was added into a Nunc™ MicroWell 96-well optical bottom assay plate (ThermoFisher) and incubated for 24 h to allow cell attachment.

For binding studies, the adherent cells were incubated with 50 μL test antibodies diluted to a final concentration of 1.0 μM in phenol red free M199/25% PBS buffer for 1 hr. at 37°C. The cells were washed in phenol red free M199 assay buffer. Secondary detection reagent AF488-conjugated AffiniPure Donkey Anti-human IgG Fc γ fragment specific antibody (1500 ng/ml Jackson Immunoresearch West Grove, PA, USA) was diluted in assay buffer then added to each well for 45 min at 37°C. The cells were washed with phenol red free M199 then stained with 0.5 μM DRAQ5™ (Cell Signaling Technology, Danvers, MA, USA) for 10 min at room temperature. The data was acquired on a mirrorball® microplate cytometer (STP Labtech Ltd., Melbourn, UK) and analyzed using Cellista software (STP Labtech Ltd) and GraphPad Prism.

### Rat Study

Male Wistar rats aged 4-6 weeks and weighing 190-230 g were purchased from Charles River (Montreal, Canada) and allowed to acclimatize to the facility for a period of at least 5 days prior to any procedure. Animals were individually housed in polypropylene cages in a 12 h light/12 h dark cycle with free access to food and water. All animal studies were approved by NRC’s Human Health Therapeutics (HHT) Research Centre Animal Care Committee and were in accordance with Canadian Council on Animal Care (CCAC) guidelines.

### *In Vivo* PK Experiments

Rats were injected with 15 mg/kg or 30 mg/kg KG207-H via lateral tail vein (*n* = 4 rats per dose). Blood was collected via the tail vein at 0.5, 1, 2, 4, 8, 24, 48, 72 and 168 hours after injection (for the 15 mg/kg dose) and 0.5, 1, 2, 6, 24, 48, 72, 96, 168, 240 and 336 hours after injection (for the 30 mg/kg dose). CSF was collected via cisterna magna puncture at 4, 8, 24, 48, 72, and 168 hours (for the 15 mg/kg dose) and 6, 24, 48, 72 and 168 hours (for the 30 mg/kg dose) after injection as described below.

### Serial CSF Collection

Rats were anesthetized with 4% isoflurane and placed in a stereotaxic frame with the head tilted downward at a 45°angle. After removing the fur and cleaning the surgical area, a midline incision was made, beginning at the occipital crest and extending caudally ~2 cm. The neck muscles were retracted to access the atlanto-occipital membrane. The dura mater overlying the cisterna magna was exposed, and a 27G butterfly needle was inserted 1 mm into cisterna magna at a sharp angle to the dura. CSF was slowly aspirated using a 1 ml syringe attached to the needle with a short piece of tubing (~15-20 μl at each collection time point). Samples were then placed in glass sample vials and frozen on dry ice. The skin was closed with sutures. The procedure was repeated up to 5 times per rat at various post-injection intervals.

### Blood Collection

Blood samples were collected from the tail vein by a 27G needle puncture and transferred to serum separator tubes (BD) and kept at room temperature for 30 min and then centrifuged at 1000 g for 10 min. The serum was transferred to sample (glass) vials and frozen on dry ice immediately.

#### Beagle Dog Study

This protocol was developed in accordance with principles of the Animals for Research Act of Ontario and the guidelines of Canadian Council on Animal Care (CCAC) and reviewed and approved by InterVivo Solutions’ (Ontario, Canada) Animal Care Committee (ACC) and by NRC’s ACC.

Sixteen healthy Beagle dogs (5 males and 11 females, ranging from 7 to 13 years) in good health were included in this parallel group, non-blinded, study. Subjects were placed into four groups matched with respect to age, body weight and gender to the extent possible. Treatment groups did not differ statistically, and groups were randomly assigned to a treatment condition (PBS or KG207-H at 5, 20 or 50 mg/kg body weight) by the drawing of lot.

Subjects received the control or test articles IV via a cephalic catheter at a rate of approximately 8-10 seconds/mL for 5-7 minutes followed by 1-3 mL of a sterile saline flush. Time 0 represented the time the entire dose and saline flush were administered.

Blood samples, approximately 3 ml, were collected via jugular venipuncture prior to dosing and at 0.25, 0.5, 1, 2, 4, and 8 hours (±3 minutes up to the 1-hour collection, ±15 minutes for remaining collections) and at 1, 2, 3, 4, 7, 8, 9, and 14 days (±1 hour) following the drug injection. Samples were transferred into a serum separator blood collection tube (SST) and allowed to clot at room temperature. The tubes were centrifuged at 2800-3200 rpm for 10 minutes at room temperature. For each sample, serum was separated equally into four cryovials.

Cerebrospinal fluid (CSF) was collected prior to dosing and at 2, 4, and 8 hours (±10 minutes), and 1, 2, 3, 4, 7, 8 and 14 days (±1 hour), following injection. Samples were obtained under short term sedation from the cisterna magna using sterile technique. Dogs were anesthetized to effect with propofol (8 mg/kg, IV) and maintained on 0.5-2% isoflurane with oxygen. Between 0.5-1.0 ml of CSF were collected at each time point. CSF were placed on wet ice until being processed by centrifugation at 3000 rpm for 2 minutes at 2-8°C in order to remove potential red blood cell contamination. Once centrifuged, CSF samples were separated into 4 equal aliquots and stored at −80°C (±5°C) until further use.

#### Drug Level Measurement in Serum/CSF Samples by Multiplexed Mass Spectrometry Analysis

FC5(H3)-hFc-ABP (KG207-H) fusion protein levels in the serum and CSF and Aβ levels in the CSF were quantified using targeted multiplexed mass spectrometry as previously described (11). Briefly, plasma and CSF samples containing KG207-H were reduced, alkylated, and trypsin digested using the previously described protocol (11, 29). For absolute quantification, calibration curves were prepared by spiking KG207-H in the appropriate matrix from naïve animals (either dog or rat plasma at 0 – 10 nmol/mL, or dog or rat CSF at 0 –20 nM) and similarly digested. All digests were analyzed on nanoAcquity UPLC (Waters, Milford, MA) coupled to ESI LTQ XL ETD mass spectrometer (ThermoFisher, Waltham, MA) in selected reaction monitoring (SRM) mode as previously described (17). The SRM method included multiplexed quantification of three peptides unique to KG207-H (FC5 ITWGGDNTFYSNSVK; hFc TTPPVLDSDGSFFLYSK; ABP + linker SLSLSPGTGGGGSGGGGSGTFGTGGASAQASLASK), one host Aβ peptide (LVFFAEDVGSNK), and one host albumin peptide (SLHTLFGDK). Isotopically heavy versions of the FC5, hFc and Aβ peptides, containing heavy C-terminus K (+8 Da), were also synthesized (New England Peptide LLC, Gardner, MA) and included as internal standards for quantification as previously described (11, 29). Raw data extraction and analysis was performed using Skyline software version 3.7 (https://skyline.ms). Lower limits of quantifications were calculated to be 0.02 nmol/mL in serum and 0.1 nM in CSF background. The albumin peptide was used to identify and exclude serum-contaminated CSF samples (11). Total Aβ levels are reported relative to the mean levels in PBS-injected animals (dog) or relative to maximum measured value (rat). During the preparation of samples for multiplexed SRM method analyses, protein samples were denatured and trypsinized to generate peptide fragments. In this process, any protein:protein complex, such as ABP:Aβ in this case, will be disrupted and thus overcomes potential interference in Aβ measurement. Thus, the described mass spectroscopy analytical method has an advantage over conventional ELISA wherein Aβ complex with other proteins might interfere with its accurate measurement unless the complex is disrupted.

#### Drug Level Measurement in Dog Serum Samples by ELISA

KG207-H levels in dog serum samples were quantified using a sandwich ELISA with FC5 capture and ABP detection. MaxiSorp ELISA plates were coated with anti-FC5 antibody (developed in-house, 1.5 μg/ml) in 20 mM Tris-HCl pH 8.0 at 4°C overnight, then blocked in 1% BSA in TBST. Appropriately diluted serum samples (in TBST) were overlaid on the plates for 1 hour. After rinsing in TBST, the wells were incubated with anti-ABP antibody (developed in-house; 1:5000 for 60 minutes at room temperature), rinsed again, and incubated with HRP-labeled anti-rabbit antibody (Jackson ImmunoResearch; 1:20,000) for 90 minutes at room temperature. Drug levels were quantified using a standard curve established by spiking TBST with KG207-H. The large number of dog serum samples necessitated use of more time- and cost-effective ELISA assay. Analysis of a subset of dog serum samples by both mass spectrometry and ELISA yielded a good correlation between KG207 measurements obtained using the two methods. Mass spectrometry was used for CSF samples since it allowed simultaneous measurements of KG207 and Aβ.

### PK Analysis

Serum and brain concentration-time profiles were analyzed using WinNonlin software (Version 8.3, Pharsight Corporation, Mountain View, CA, USA). Serum concentration-time data for KG207-H were analyzed using naive pooled data with two-compartment model, IV administration (bolus for rat, short infusion for dog), first-order elimination, and macro-rate constants to estimate the following pharmacokinetic parameters: Volume of distribution of the central compartment (V_1_) and of the peripheral compartment (V_2_), Clearance (CL), Inter-compartmental Clearance (CL_D_) and overall elimination half-life (t_1/2β_). Overall, the goodness of fit was based upon the predicted estimate and percent coefficient of variation (% CV) for primary and secondary parameters, as well as inspection of residual plots between observed and predicted concentration-time data.

A non-compartmental approach consistent with IV administration and linear/log trapezoidal method was employed to estimate the area under the curve (AUC) of the serum concentration *vs* time and CSF concentration *vs* time. For CSF, the AUC from the start of dose administration to the last observed quantifiable measurement (AUC_0-168_) was estimated. Estimation of average concentration ratios for AUC_0-168_ is reported as (AUC_CSF, 0-168_ /AUC_Serum, 0-168_) × 100.

## RESULTS AND DISCUSSION

**Fig. 1:**
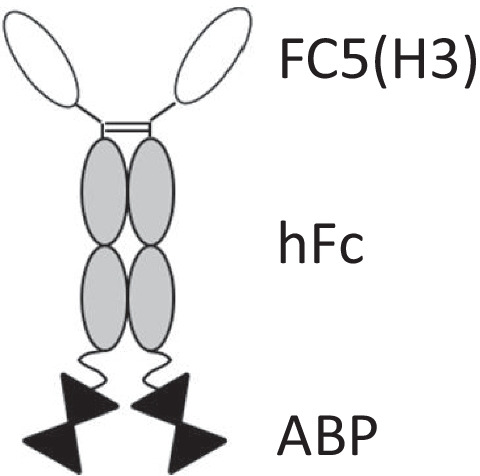
Schematic representation of KG207-H. FC5 (H3): humanized blood-brain barrier crossing single-domain antibody; hFc: human IgG1 Fc fragment; and ABP: amyloid-β binding peptide.

The design of FC5(H3)-hFc-ABP (KG207-H) fusion protein, comprising humanized single-domain camelid antibody FC5, Fc fragment derived from human IgG1 to enhance serum half-life and amyloid-β oligomer binding peptide ABP, is schematically represented in Fig. 1. The fusion protein was expressed and produced in CHO^BRI^ stable cell line and purified by Protein A affinity column and cation exchange (CEX) chromatography.

**Fig. 2:**
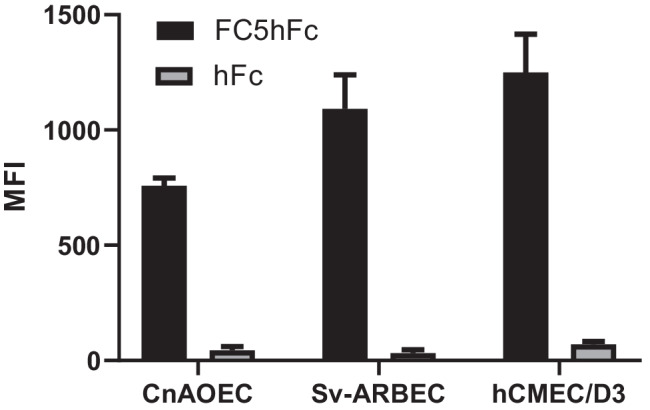
FC5 binding to canine, rat and human endothelial cells. Binding studies were carried out as described in Methods section. Bar graph represents data from two independent studies and expressed as mean and range. CnAOEC: Canine (dog) aortic endothelial cells; SvRBEC: rat brain endothelial cells; hCMEC/D3: human brain endothelial cells. MFI: Mean Fluorescence Intensity. Since the objective of this experiment was simply to demonstrate cross-reactivity of FC5 with canine endothelial cells as a prerequisite for PK studies, binding across the three cell types was not compared statistically.

Comparative inter-species studies require a confirmation of the species cross-reactivity of the antibody binding to its antigen. FC5 has been shown previously to be broadly species cross-reactive across rodent, NHP and human antigen. In this study, we compared FC5 binding to rat brain endothelial cells (svRBEC), human brain endothelial cells (HBEC/hCMEC-D3) and dog aortic endothelial cells (CnAOEC). FC5 bound dog aortic endothelial cells in both adherent (Fig. 2) and suspension formats (data not shown), similar to the observed binding to rat and human BEC (Fig. 2). Although dog endothelial cells were from the aorta and not from the brain, these results suggested that FC5 demonstrates a rat-canine endothelial receptor cross-reactivity and could be used in comparative PK studies in rat and dog. TMEM30A, a putative receptor for FC5, is expressed in peripheral vessels, and is comparatively enriched in brain cerebrovascular endothelial cells from different species (30, 31). There is strong evidence of TMEM30A expression in human, mouse and dog aorta (publically available data from https://www.ncbi.nlm.nih.gov/geo/query/acc.cgi?acc=GSE199709; https://www.ncbi.nlm.nih.gov/geo/query/acc.cgi?acc=GSE193275). In dog aorta, TMEM30A is expressed with ranking in the top 50% by abundance (32). Thus, use of dog aortic endothelial cells for FC5 binding in this study is justified as dog brain endothelial cells were not available.

**Fig. 3:**
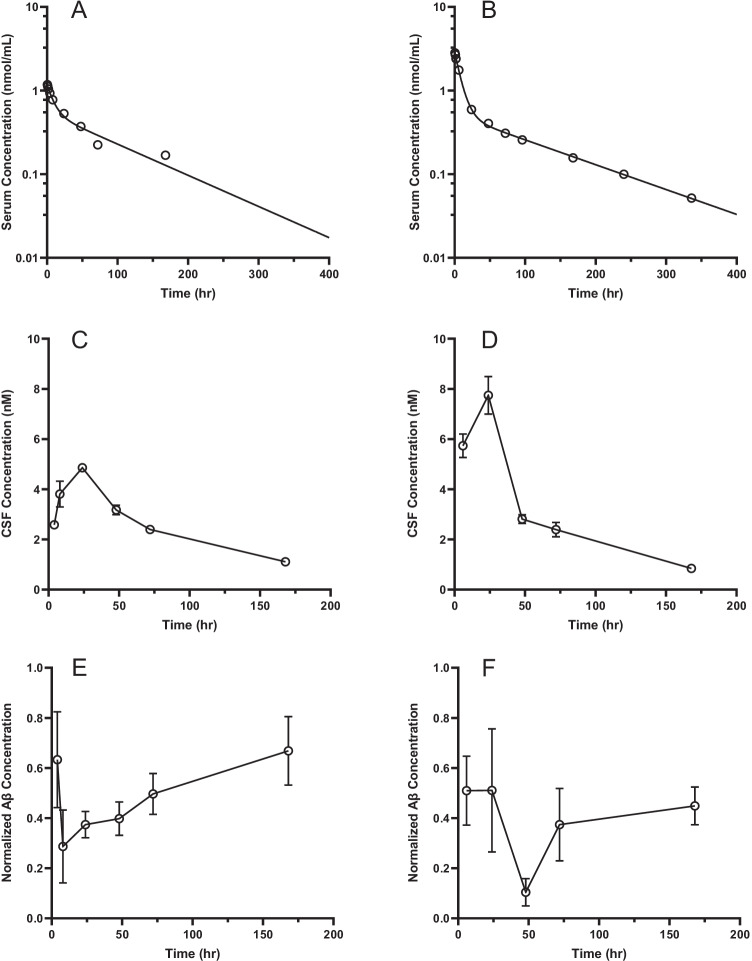
Serum KG207-H [A, B, Observed (o) *vs* predicted (−)], CSF KG207-H (**c**, **d**) and CSF Aβ (relative to maximum concentration, **e**, **f**) concentration-time profiles following a 15 mg/kg (**a**, **c**, **e**) or a 30 mg/kg (**b**, **d**, **f**) intravenous bolus administration of KG207-H to naïve rats, Mean ± SEM (n = 4).

**Fig. 4:**
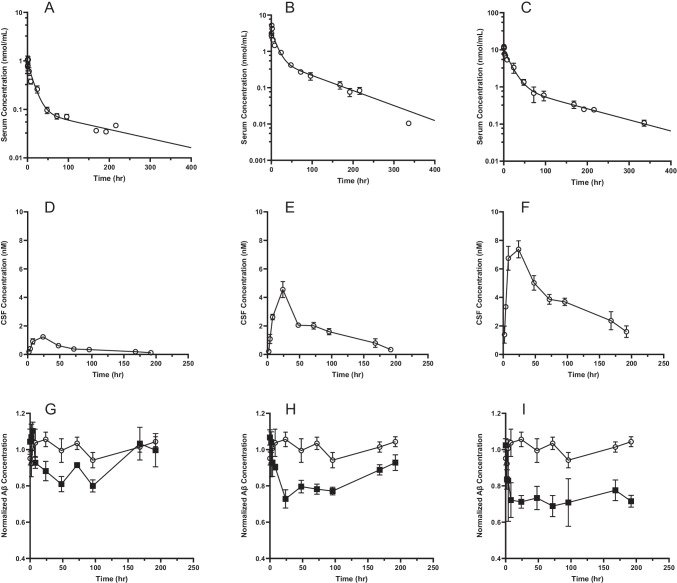
Serum KG207-H [**a**, **b**, **c**, Observed (o) *vs* predicted (−)], CSF KG207-H (**d**, **e**, **f**) and CSF Aβ (**g**, **h**, **i**; PBS control: open symbols; KG207: closed symbols) concentration-time profiles following a 5 mg/kg (**a**, **d**, **g**), 20 mg/kg (**b**, **e**, **h**) or 50 mg/kg (**c**, **f**, **i**) short intravenous infusion of KG207-H to naïve dogs. Observed concentrations reported as mean ± SEM (*n* = 3-4).

The serum PK profiles of KG207-H following IV administration in rats and dogs are shown in Figs. 3 and 4. The serum drug concentration *vs* time profiles exhibited a biphasic decrease which are fitted to a two compartment PK model at all administered doses. The serum PK parameters are summarized in Table I. In both species, the C_max_ values appear to increase proportionally with the administered dose. At doses tested in this study, the clearance values of KG207-H were slightly higher in rats compared to dogs. Relatively consistent clearance among all groups suggested linear pharmacokinetics at selected dose range (Table I). These clearance values are higher than those of a typical non-targeting mAb but lower than a typical sdAb without Fc domain (33–35). Target of FC5, TMEM30A (Cdc50a) is expressed in both brain- and peripheral endothelial cells, which likely contributes to the faster systemic clearance of KG207-H compared to a monoclonal antibody which does not bind any peripheral target.

**Table I:** Mean PK Parameter Estimates (%CV) from 2-Compartment Analysis of Serum Concentration-Time Profiles After Intravenous Bolus Administration of KG207-H to Naïve Rats or Dogs

		Rat		Dog		
Parameter	Unit	15 mg/kg	30 mg/kg	5 mg/kg	20 mg/kg	50 mg/kg
*C*_*max*_	nmol/mL	1.2 (1.6)	2.9 (2.6)	0.9 (7.5)	3.7 (8.4)	10 (8.0)
*t*_*1/2β*_	hr	80.8 (17.6)	101 (3.0)	160 (38)	73 (11)	101 (12)
*CL*	mL/hr./kg	2.5 (9.7)	3.6 (1.5)	1.8 (15)	2.4 (5.3)	1.9 (5.2)
*CL*_*D*_	mL/hr./kg	6.4 (13.3)	7.4 (7.1)	2.3 (18)	2.2 (25)	1.2 (21)
*V*_*1*_	mL/kg	143 (1.6)	116 (2.6)	62 (11)	62 (8.4)	57 (8.0)
*V*_*2*_	mL/kg	122 (18)	293 (3.7)	208 (34)	104 (15)	97 (14)
*AUC*	nmol*hr./mL	69 (9.7)	96 (1.5)	32 (15)	95 (5.3)	299 (5.2)

*C*_*max*_*: Predicted peak concentration. t*_*1/2β*_*: Terminal half-life. CL: total body clearance*. *CL*_*D*_*: Intercompartmental clearance. V*_*1*_*: Central compartment volume of distribution. V*_*2*_*: Peripheral compartment volume of distribution. AUC: predicted area under the plasma concentration time curve from time 0 to infinity*

Volume of distribution appears to be higher than that of a typical mAb, as expected from a construct with a lower molecular weight (87.7KD). With the exception of the 5 mg/kg group in dogs that had a longer terminal half-life (160 hr), the values obtained in all other groups were similar and ranged between 73 to 101 hr. The longer half-life observed in dogs at 5 mg/kg might have resulted from the missing data-point at 336 h which could lead to the less precise estimate of this secondary parameter (also indicated from the high % CV associated with this value). Despite the slightly higher apparent volume of distribution in rat compared to dog, the PK characteristics were relatively similar between these two species.

KG207-H progressively entered the CNS and had reached the maximum CSF concentration at around 24 hours after IV bolus injection, in both rat and dog (see Figs. 3 and 4 and Table II). The concentration-time curves in serum and CSF were parallel after the C_max_ was reached in the CSF. The half-lives in CSF (73-107 hrs) approximated their serum half-lives for most of the dose groups (73-101 hrs). In rats, the ratios of CSF AUC_0-168_ over serum AUC_0-168_ were slightly higher at 15 mg/kg when compared to 30 mg/kg. However, on average, ratios of CSF AUC_0-168_ over serum AUC_0-168_ were fairly consistent, ranging from 0.3% to 0.5%. These ratios were higher than the apparent CNS exposure of most biologics which is typically around 0.1%. (10, 36, 37).

**Table II:** Mean ± SD AUC_*0-τ*_ of the CSF Concentration-Time Profiles and the Ratio Over AUC_*0-τ*_ of the Serum Concentration-Time Profiles Following Intravenous Bolus Administration of KG207-H to Naïve Rats or Dogs

		Rat		Dog		
CSF/Serum AUC ratios	Unit	15 mg/kg	30 mg/kg	5 mg/kg	20 mg/kg	50 mg/kg
*C*_*CSF max*_	nmol/L	4.5 ± 0.9	7.7 ± 1.5	1.2 ± 0.2	4.0 ± 1.4	7.4 ± 1.0
*CSF AUC*_*0-168*_	nmol*hr./mL	0.40 ± 0.05	0.47 ± 0.06	0.09 ± 0.01	0.32 ± 0.05	0.72 ± 0.11
*CSF AUC*_*0− 168*_/ *Serum AUC*_*0− 168*_	%	0.73 ± 0.08	0.50 ± 0.07	0.38 ± 0.03	0.35 ± 0.06	0.28 ± 0.03

*AUC*_*0-168*_*: area under the plasma concentration time curve from time 0 to 168 hours. C*_*CSF max*_*: Observed KG207 maximum concentration in CSF*

Similar to findings in the current study, peak CSF concentrations of FC5-enabled peptides (10) and FC5-full IgG antibodies (13) occurred only around 24 hours after systemic injection in rats. Here we report an equivalent delay in CSF C_max_ in the dog.

In the absence of active transport, antibody entry into CSF will be highly restricted by the choroid plexus (CP) epithelial blood-CSF (BCSFB) barrier, which is less tight than the endothelial BBB. Based on recent microdialysis experiments by Chang *et al.*, it was observed that C_max_ of antibodies crossing BCSFB via a non-specific permeation in the CSF occurs relatively fast after intravenous bolus administration and then decays in parallel with the serum concentrations (38). Actively transported antibodies could cross either BBB or BCSFB or both, provided that the RMT receptor is expressed in BBB endothelial cells or in choroid plexus epithelium or both; for example, TfR antibodies undergo RMT at both BCSFB and BBB interfaces (39). Both increased brain:plasma and CSF:plasma ratios of the TfR-targeting antibodies compared to non-transporting antibodies (Nip228) has been observed in rats (36). This is in contrast to Kariolis *et al*. (40), where BACE-1 antibody functionalized with the TfR-binding ATV showed increased parenchymal, but not CSF levels in mice and non-human primates. More importantly, in our studies with FC5 as BBB carrier, we have observed both increased brain: serum and CSF: serum ratios of FC5-fused cargos compared to control (non-transported) antibody (13; Supplementary Fig. 1). In the current and our previous studies with KG207 (17), the C_max_ in the CSF was consistently delayed by approximately 24 h compared to C_max_ in the serum. Direct measurements of FC5-fusion protein(s) in the rat brain (10, 13) and KG207 in both total brain homogenates and vessel-depleted brain parenchyma in the mouse (Supplementary Figs. 1 and 2) showed increased levels of KG207 in the brain parenchyma at both 4 h and 24 h after administration compared to respective non-BBB permeable control V_H_H construct. Based on these results we interpret the delay in the CSF C_max_ observed in dogs to mean that KG207-H in the CSF derives mainly from the brain interstitial fluid (and thus reflects true BBB penetration), rather than resulting solely from a direct entry across the BCSFB barrier.

KG207-H achieved comparable CNS/serum exposure ratios in rats compared to those observed with the rat-TfR-binding antibody (OX26) affinity variants (36). However, the serum half-life of KG207-H is longer (~2-4 fold) when compared to medium affinity OX26 variants (OX26-76 and OX26-108) (36). High TfR enrichment in reticulocytes (bone marrow) is responsible for a rapid serum clearance of higher affinity TfR antibodies. Therefore, long serum half-life of KG207-H in both rodents and dogs could extend the CNS exposure and potentially the duration of action compared to TfR antibodies.

**Fig. 5:**
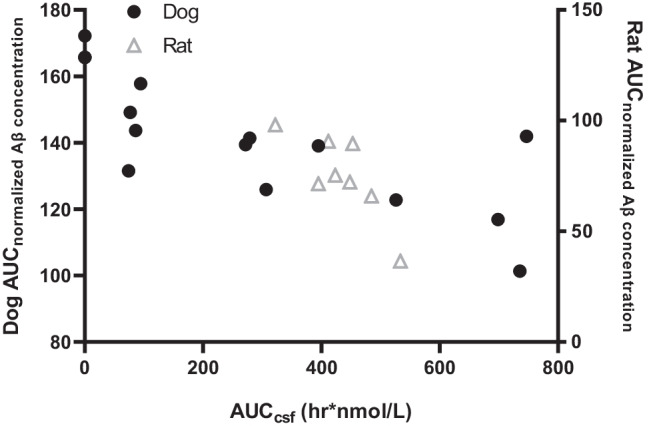
AUC_0-168_ values of normalized Aβ concentration *vs*. CSF AUC_0-168_ values of KG207-H following a short intravenous infusion to naïve dogs (5 mg/kg, 20 mg/kg or 50 mg/kg) or an intravenous bolus administration to rats (15 mg/kg or 30 mg/kg).

As indicated in Fig. 5, there is an inverse relationship between the apparent CSF KG207-H exposure (CSF KG207-H AUC_0-168_) and the Aβ level (CSF Aβ AUC_0-168_) suggesting target engagement by KG207-H within CNS. The wildtype rat used here, with endogenous expression of Aβ and no Aβ pathology, showed a high level of variability in CSF Aβ levels in comparison to transgenic rats overexpressing mutant APP (17). Similarly, substantial inter-individual differences in CSF Aβ levels in Beagle dogs have been reported, even in young to middle-aged animals (41). To account for run-to-run variability in the analytical method used, data from each KG207-treated dog in this study were expressed relative to the PBS-injected dog analyzed in the same analytical batch (Supplementary Fig. S3).

Due to some inter-individual variability in the response to treatment and relatively small dataset, we were not able to derive an indirect response PK/PD model that describes the relationship between KG207-H and Aβ concentrations in the CSF with sufficient level of confidence. Nevertheless, the reduction of CSF Aβ in response to KG207-H appears to be consistent across species, and is inversely related to the level of KG207-H exposure in CSF. Notably, as seen in Fig. 3 increasing the dose of KG207-H in dog did not result in greater maximum effect on CSF Aβ, but rather prolonged the duration of the maximum effect. Thus, the present study confirms that FC5 is capable of delivering ABP to the CNS in both rat and dog, and, that having gained access to CNS, ABP is capable of targeting Aβ.

We have shown previously that ABP binds full-length Aβ_1-42_ as well as Aβ_17-42_, suggesting that the ABP binding domain on Aβ is downstream of amino acid 17 (Supplementary Fig. S4). Consistent with the above observation, we have shown that ABP reduces cellular toxicity induced by both Aβ_1-42_ and Aβ_25-35_ (15). This middle to C-terminus region is conserved in both rat and human Aβ peptide, suggesting that ABP likely binds rat Aβ sequence. Although some reports suggest that rat Aβ aggregates, but does not form fibrils, *in vitro* and *in vivo* under certain conditions (42, 43), rat Aβ is not believed to aggregate and accumulate in the form of amyloid plaques in wt rat. Therefore it is not clear whether the decrease in CSF-Aβ in rat is due to binding of ABP to aggregated Aβ in this model.

The decrease in rat/dog CSF Aβ could be caused by a shift in the concentration equilibrium between blood and CSF due ABP binding/clearance of circulatory Aβ. In a separate study in dogs, we compared non-BBB permeable fusion protein A20.1-mFc-ABP (A20.1 is control V_H_H that does not bind any mammalian target) to BBB-permeable KG207-M (FC5-mFc-ABP). Although serum PK profiles for both constructs were similar after intravenous injection, KG207-M appeared in the CSF at much higher levels compared to A20.1-mFc-ABP and, in contrast to A20.1-mFc-ABP, reduced CSF Aβ (Supplementary Fig. S5). These findings suggest that KG207-M effect on CSF-Aβ was not due to ABP binding to circulating Aβ since BBB-impermeable A20.1 construct with functional ABP did not affect CSF-Aβ (Supplementary Fig. S5).

While the finding of lowered CSF Aβ in this study cannot be directly extrapolated to the brain, it is worth noting that reduced CSF Aβ observed in transgenic rats after KG207-M treatment was accompanied by significantly diminished brain amyloid (17). The relationship between the biomarker and drug concentration aligns with a typical indirect response model with stimulation of Aβ loss by drug concentration. The mechanism by which KG207-H clears Aβ is multifactorial and could not be incorporated in the model at present time.

Based on a detailed mechanistic model of the effect of Aβ-targeting therapeutics on soluble Aβ dynamics in the brain, CSF and plasma, it has been concluded recently that the transport across the BBB is likely a limiting factor in Aβ target engagement and reduction in AD (44). The model predicts that enhancing the brain exposure of a drug would have a much more profound effect on Aβ reduction than increasing its affinity to Aβ, underscoring the importance of engineering BBB permeability into AD therapeutics (44). The enhanced CNS penetration of KG207-H relative to anti-Aβ antibody therapeutics and the translation of the serum/CSF PK and PD profiles of KG207-H from rat to the larger and more complex dog brain highlights the utility of FC5 as a BBB carrier to improve brain exposure of CNS-targeted therapeutics.

Aged dogs, particularly Beagles, have been used as preclinical models to study PK/PD of therapeutics targeting AD as they are phylogenetically more closely related to humans compared to rodent, with high anatomical and physiological similarities (45). Additionally, with their larger body and brain sizes, which allows for larger sampling volumes, dogs appear to be appropriate models for biomarker studies in the brain (45). Canines, like NHP, may also better represent human populations than the inbred rodent model due to their genetic heterogeneity. Furthermore, pre-clinical studies with Beagle dogs appear to predict the efficacy of some AD therapeutics in humans (46, 47). Thus, the current study, which demonstrates a good translatability in brain exposure between rodent and dog, strongly supports further development of KG207-H as a potential AD therapeutic.

## CONCLUSION

The results of the current study show that the humanized BBB carrier FC5 is capable of delivering the therapeutic Aβ-targeting payload ABP into the CNS of both rodent and dog, and that the delivered ABP can effectively engage target Aβ. The strong similarities in the results between a rodent model with smaller and less complex brain and a dog model with larger and more complex brain demonstrates translational attributes of FC5. In conclusion, this study greatly aids in de-risking further preclinical and clinical development of KG207.

## Supplementary Information

ESM 1 (DOCX 158 kb)
