# Supplementary material for: Pharmacokinetics and Pharmacodynamic Effect of a Blood-Brain Barrier-Crossing Fusion Protein Therapeutic for Alzheimer’s Disease in Rat and Dog
Source: Pharm Res. 2022 Jun 15;39(7):1497–507. doi: 10.1007/s11095-022-03285-z (PMC9246806; doi:10.1007/s11095-022-03285-z)
Supplement: Supplementary file 1 — (DOCX 158 kb) [file 11095_2022_3285_MOESM1_ESM.docx]

Supplemental Material:

**Figure S1:** **Increased brain-delivery of KG207-H carrying BBB-permeable FC5 compared to non BBB-permeable A20.1-Fc.** Transgenic mice were intravenously injected with 15 mg/kg of either A20.1-Fc or KG207-H, and CSF and brains were collected after 24h. Levels of A20.1-Fc (grey bars) and KG207-H (red bars) in the CSF and perfused brain were measured by nanoLC-SRM analysis in at least 4 animals. Bars represent mean and SD. Although A20.1-Fc did not carry any payload in this study, similar results were observed with A20.1-mFc2a-ABP and FC5-mFc2a-ABP (KG207-M, data not shown) wherein both constructs carried ABP payload.

**Figure S2: Time-dependent appearance of KG207-H in brain parenchyma.** Tg mice were intravenously injected with either PBS or 15 mg/kg of KG207 and perfused brains were collected at 4 or 24h. The brains were separated into vessel and parenchyma fractions and analyzed by nanoLC-SRM to simultaneously quantify Glut1, GFAP and KG207 levels in a multiplexed manner. A) Normalized levels of Glut1/Slc2a1, a vessel marker, in each brain fraction to demonstrate success of vessel depletion from parenchymal samples. Most of the signal in parenchyma samples is in the background (< or below limit of detection LOD). B) Normalized levels of GFAP, a parenchymal marker, in each brain fraction (vessel, parenchyma or naïve mouse whole brain) to confirm presence of brain proteins in parenchymal samples. Levels in A and B are normalized to the levels in the naïve whole brain (not shown). C) Absolute levels of KG207 in each brain fraction. Levels were quantified using standard curves and QC (not shown). PBS-injected animals showed no antibody signals (<) in either fraction since they were not injected with KG207.

**
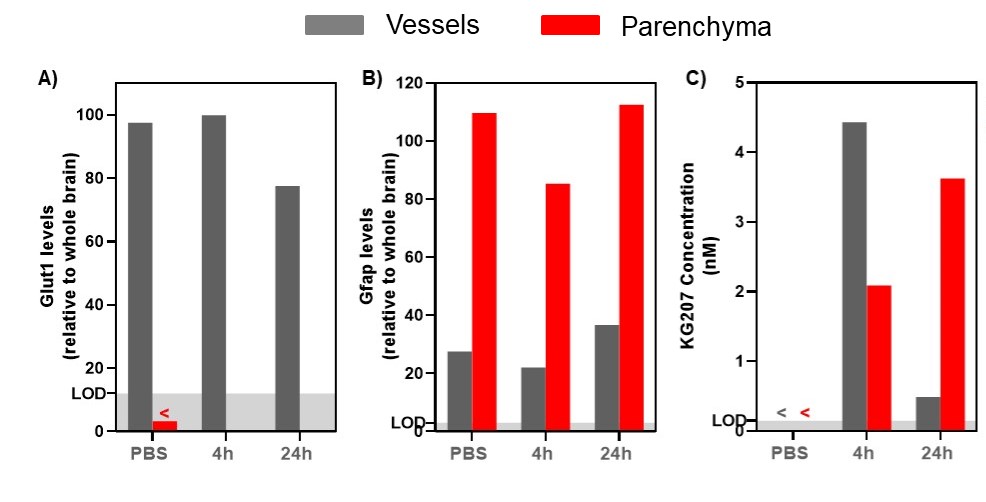
**

**Figure S3.** Animal-to-animal variability in total amyloid beta measurements in dog CSF samples examined by LC-MRM before drug (KG207-H) administration. Shown are MRM intensities corresponding to amyloid beta peptide-specific signals in CSF samples from the treatment group before dosing (grey bars). Since samples were run in different batches (days-to-weeks apart), a higher variability was seen among different batches than within each batch. In order to compare among all batches. The results for the drug (KG207-H)-treated CSF samples presented in the manuscript were normalized to the CSF levels in PBS-injected animals prior to dosing (black bars)

**Figure S4: Amyloid-β binding peptide (ABP) binds synthetic Aβ_1-42_ peptide equally well as synthetic Aβ_17-42_. A.** ABP (500ng) was coated on ELISA plates, incubated with aggregated Aβ peptides at 200 nM for 45 min at room temperature and bound Aβ was detected with 4G8 antibody that recognizes Aβ sequence 17-24 as described (Chakravarthy et al., 2013). **B**. Aß_1-42_ and Aß_17-42_ were doted on nitrocellulose paper at the indicated amounts, incubated with ABP peptide (200nM) and bound ABP was detected with ABP-selective antibody as described (Chakravarthy et al., 2013). These results suggest that P4-5 binding motif is downstream of amino acid 15 towards the C-terminus.


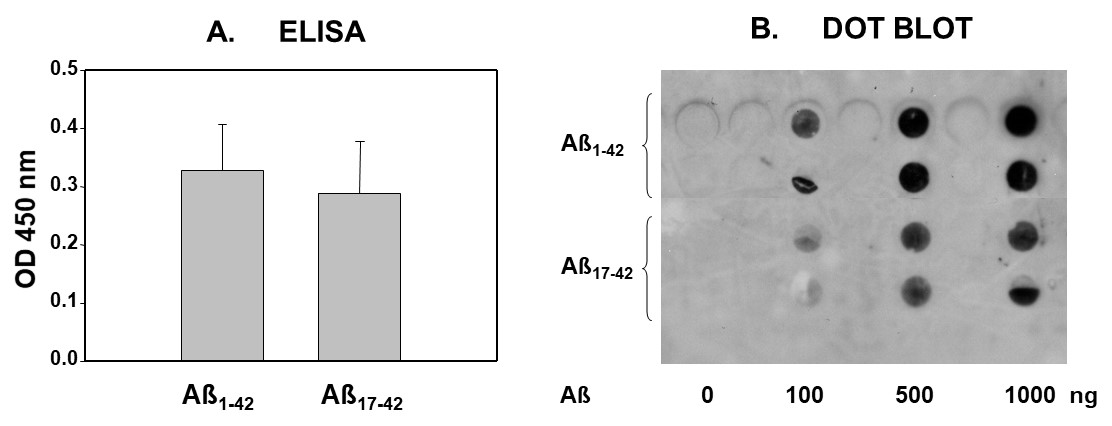


**Figure S5:** Levels of A20.1-mFc-ABP or FC5-mFc-ABP or total amyloid beta in serum and CSF 4h or 24 h after intravenous injection of 15 mg/kg of either fusion protein in dogs. Absolute levels of A20.1-mFc-ABP and FC5-mFc-ABP were quantified in serum and CSF using LC-MRM analysis. Total amyloid beta was analyzed simultaneously in the CSF samples in a multiplex manner. Asterisks (*) indicates levels below limits of detection at time 0 h.
